# The multiple facets of drug resistance: one history, different approaches

**DOI:** 10.1186/1756-9966-33-37

**Published:** 2014-04-28

**Authors:** Evandro Luís Niero, Bianca Rocha-Sales, Camila Lauand, Beatriz Araujo Cortez, Marcelo Medina de Souza, Paula Rezende-Teixeira, Marcel Shiniti Urabayashi, Adam Arai Martens, Jorge Henrique Neves, Gláucia Maria Machado-Santelli

**Affiliations:** 1 Department of Cell and Developmental Biology, Institute of Biomedical Sciences, University of São Paulo, Av. Prof. Lineu Prestes, 1524, Cidade Universitária, 05508-000 São Paulo, SP, Brazil

**Keywords:** Chemoresistance, Multidrug resistance, Cell death, 3D cell culture, Cancer stem cells

## Abstract

Some cancers like melanoma and pancreatic and ovarian cancers, for example, commonly display resistance to chemotherapy, and this is the major obstacle to a better prognosis of patients. Frequently, literature presents studies in monolayer cell cultures, 3D cell cultures or *in vivo* studies, but rarely the same work compares results of drug resistance in different models. Several of these works are presented in this review and show that usually cells in 3D culture are more resistant to drugs than monolayer cultured cells due to different mechanisms. Searching for new strategies to sensitize different tumors to chemotherapy, many methods have been studied to understand the mechanisms whereby cancer cells acquire drug resistance. These methods have been strongly advanced along the years and therapies using different drugs have been increasingly proposed to induce cell death in resistant cells of different cancers. Recently, cancer stem cells (CSCs) have been extensively studied because they would be the only cells capable of sustaining tumorigenesis. It is believed that the resistance of CSCs to currently used chemotherapeutics is a major contributing factor in cancer recurrence and later metastasis development. This review aims to appraise the experimental progress in the study of acquired drug resistance of cancer cells in different models as well as to understand the role of CSCs as the major contributing factor in cancer recurrence and metastasis development, describing how CSCs can be identified and isolated.

## Introduction

The use of chemical agents to treat patients with cancer began with two studies in the 1940s. The synthesis and application of nitrogen mustard, a derivate compound from the chemical warfare agent mustard gas which, in addition to other damages, causes injury to blood cells and bone marrow degeneration. At the end of the same decade, observation of the relationship between folic acid and leukocyte proliferation and synthesis of compounds with antagonistic action to folic acid promoted a breakthrough in cancer treatment.

In 1942, Alfred Gilman and Louis Goodman treated a patient with advanced lymphosarcoma, which no longer responded to radiotherapy or surgery, with nitrogen mustard. Halfway treatment they could perceive a symptomatic improvement and, at the end of the treatment period, biopsy revealed no tumor. However, the tumor reappeared weeks later and the treatment did not cause the same effect, with the patient’s death weeks later [1]. In another clinical research, Sidney Farber introduced the administration of folic acid antagonists in patients with acute leukemia. The results showed that some compounds have the effect of temporarily inhibiting cell proliferation [2].

The results obtained in these two studies were similar in some aspects, such as the chemical agents being able to kill cancer cells, causing side effects and reappearance of tumors exhibiting resistance to the initial treatment. Subsequently to these events, other research groups sought to find substances that combined a more effective action on cancer cells and fewer side effects, as well as new strategies for drug administration [3-6].

Thus, drug resistance of cancer cells has been the subject of intense study. One of the first studies specifically investigating the resistance to folic acid antagonists was performed by Law [7]. Based on a study of bacterial resistance to a virus [8], the study of Law was conducted with the goal to find out the source of resistance of leukemic cells to folic acid antagonists, coming to the hypothesis that resistance seemed to arise from random mutations and selection.

The resistance of tumor cells to cytotoxic drugs is the major cause of failure of chemotherapy. This resistance, intrinsic or acquired, is a reflex of the result of numerous genetic and epigenetic alterations in cancer cells [9-11].

Anticancer drugs have targeted mainly the DNA, activating or silencing gene expression, and to do so drugs must penetrate an important cellular barrier, the plasma membrane. This should work as a line of defense and physical resistance to many classes of drugs [12,13]. Specific carriers actively transport some drugs through the membrane, and the resistance to them can be generated by reducing the drug carrier affinity or by decreasing the speed of transport. However, the efflux mechanism is assumed as the main responsible for the multiple drug resistance phenotypes.

This type of resistance involves the participation of the mechanisms of multidrug resistance (MDR), which include P-glycoprotein (P-gp), belonging to a family of ATP-dependent transporters. The intrinsic resistance is characterized by lack of sensitiveness to drug since the beginning of treatment, which is directly involved in the drug efflux [14].

Acquired resistance can develop by continuous exposure to drugs, which can trigger different cellular responses, such as blockage of apoptotic pathways, increased ability to repair DNA, changes in the control points of the cell cycle or induction of specific genes.

In the 40’s, nitrogen mustard gas was used as a cytotoxic agent. Twenty years later, anticancer drugs derived from natural products (e.g. vinca alkaloids) have emerged, and these drugs were more harmful against tumor cells. Nevertheless, until the present days, the search for effective cancer therapies persists.

Nowell [15] proposed that the tumor develops from a single cell clone, which acquires selective advantage over the normal cell that gave rise to it. This hypothesis was supported by some evidence in common: biochemical or cytogenetic. Actually, in many primary tumors, cells exhibit the same abnormal karyotype. Nowell’s proposal gave rise to what is known today as the clonal evolution model of a tumor cell population, in which natural selection in tumors leads to evolutionary changes and possibly drug resistance, ensuring the survival of cancer.

Another possibility for the development and maintenance of cancer arose when Lapidot *et al.*[16] and later Bonnet and Dick [17] found a subpopulation of cells in human chronic myeloid leukemia, which was capable of developing the disease in Severe Combined Immunodeficiency or Non-Obese Diabetic/Severe Combined Immunodeficiency mice (SCID or NOD/SCID). These cells showed a phenotype to surface receptors (CD34^+^/CD38^-^) similar to hematopoietic progenitor cells and the same ability to self-renewal and differentiation, so they were termed as cancer stem cells (CSCs). It was also confirmed the presence of CSCs in several solid tumors (reviewed by Visvader and Lindeman [18]).

The purpose of this review is to report the main molecular mechanisms that lead to drug resistance. This article also aims to show some new methods used to study drug resistance in 2D and 3D cell cultures, as well as to understand the role of CSCs as the major contributing factor in cancer recurrence and later metastasis development, describing how CSCs can be identified and isolated.

## Review

### Drug resistance

Several mechanisms are associated with chemoresistance of tumor cells, but two of them have been extensively investigated along the years: apoptosis inhibition [11,19-21] and multidrug resistance, which is responsible for exporting cytostatic substances through the cell membrane [22-25].

#### Multidrug resistance

Various factors can contribute to chemoresistance in tumors, such as the cellular microenvironment and some molecules synthesized by these cells [23]. The ABC (ATP-binding cassette) protein superfamily plays an important role in the distribution of intrinsic and extrinsic (drugs, for example) molecules to the human organism. Internalization of these substrates (molecules, drugs) occurs by active transport. Their transport is dependent on the hydrolysis of ATP. Members of that superfamily of proteins are expressed in many tissues and their isoforms are widely studied. Among them we can mention the group of MDR proteins [26]. In 1987, researchers have shown that P-glycoprotein, one of the most important member of the ABC transporter superfamily, is also encoded in normal tissues; they used the monoclonal antibody MRK16 to determine the location of P-gp [27]. Cancer cells show different expression of MDR proteins and this is a huge contributor to chemoresistance in tumors [23].

The main form of drug resistance is the capacity of cells to express genes that encode membrane transport proteins [22] as P-gp, encoded by ABCB1 (MDR1) gene. The proteins that form the MDR system are able to alter the efflux and influx of many drugs, and so, change the cytotoxic effects of these drugs [23]. Many chemotherapeutic drugs, such as anthracyclines, are substrate for the MDR proteins, and this could impair the effectiveness of cancer treatment [28-30].

Aran *et al.*[24] observed that NIH3T3 (fibroblast of *Mus musculus* embryo) cells were positively influenced when treated with colchicine. Higher concentrations of colchicine increased the expression of the MDR1 gene that encodes P-glycoprotein, so an augment of the drug would be directly related to drug resistance.

Januchowski *et al.*[22] studied six ovarian cancer cell lines W1MR, W1CR, W1DR, W1VR, W1TR and W1PR (respectively resistant to methotrexate, cisplatin, doxorubicin, vincristine, topotecan and paclitaxel – that is the most commonly prescribed drug to the treatment of ovarian cancer). The W1 cell line was previously established by their group years before. It is important to note that the tissue was obtained from an untreated patient and the resistant cell lines were obtained by exposure of the W1 cell line to increasing concentrations of each drug. Their results showed high levels of P-gp protein expression in W1PR cell line, pronounced expression in W1DR and low levels in W1VR, compared to others cell lines that did not express P-gp, The results suggest that P-gp is the responsible for chemoresistance in these cell lines. The authors also found a relation between the MRP2 transcript level and methotrexate resistance in the cell lines described above.

Usually, cancer treatment combines surgery and chemotherapy/radiotherapy in order to improve patient survival or eradicate the disease. Oosterwijk *et al.*[31] concluded that it is possible to sensitize chondrosarcoma established cell lines and primary cultures to doxorubicin and cisplatin by repairing the apoptotic machinery.

Although there are many drugs that can act on P-gp to circumvent drug resistance in chemotherapy, their effective action can be compromised due to the multiplicity of signal transduction pathways involved in P-gp-mediated MDR, such as MAPK, JNK, PI3K, among others; as well as some transcription factors, like NF-κB, TNF-α, PTEN that could confer different levels of P-gp expression in different environments and conditions (reviewed in Sui *et al*. [32]).

Besides P-gp, another protein that is widely investigated is the MRP1 (multidrug resistance associated protein 1). This protein is greatly related to chemoresistance in different types of tumors, such as lung cancer, but its expression is a characteristic of childhood neuroblastoma [25].

Increased expression of MRP1 is strongly associated with the capacity of cancer cells to migrate and form a secondary tumor [33]. Other studies have shown that MCF-7 cell line cultured as spheroids exhibited an increased resistance to doxorubicin and cell-cell interactions could be significant modulators in the drug resistance inMCF-7 cell line and a resistant variant (MDR-MCF-7) [34]. These data indicate a link between MDR and tumor ability of invasiveness and metastasis.

Micro RNAs (miRNA), a family of small noncoding RNAs that regulate gene expression, can be involved in chemotherapy resistance through the regulation of MDR proteins at a post-transcriptional level. The interaction of miRNAs with the targeted mRNA can negatively modulate MDR proteins improving the tumor cell response to anticancer drugs. miRNAs are heavily explored because they represent an alternative for combined therapeutic of cancer. Yang *et al.*[35] described that miR-223 can downregulate ABCB1 and mRNA levels, suggesting that miR-223 plays an important role in the regulation of MDR proteins mediated by ABCB1 gene product in HCC cell lines.

The gene ABCC4 encodes the MRP4 protein, which is found in many tissues like renal tubules and blood cells and is another efflux membrane transporter. miR-124a and miR-506 significantly decreased MRP4 protein levels in HEK293T/17 (normal human embryonic kidney), however these miRNAs did not change the gene transcription levels [36]. MCF7 mitoxantone-resistant cells (MCF7/MX) derived from MCF7 cells, overexpress the breast cancer resistance protein (BCRP), encoded by the ABCBG2 gene, which is a target of miR-181a. The induction of miR-181a overexpression increased the sensitivity of both lines, MCF7 and MCF7/MX, to mitoxantone [37].

#### Cell death and chemoresistance

The drug-activated cell death pathway depends on the cell type. Thus, a chemotherapeutic substance may trigger a large variety of tumor responses according to the organ or tissue considered. Many of the signals that elicit apoptosis converge on the mitochondria, which respond to pro-apoptotic signals by releasing cytochrome c [38]. There are two great classes of chemotherapeutic drugs: molecules that induce cell death in interphase cells, frequently by causing DNA damage, like cisplatin [39,40] (reviewed in Eckstein [41]), and others that induce cell death by mitosis blockage, usually by interfering in microtubules dynamic, like paclitaxel [42,43].

p53 was the first described tumor suppressor gene associated with apoptosis and it has been extensively studied along the years, because mutations in this gene occur in the majority of human tumors. Furthermore, p53 mutations are frequently associated with advanced tumor stage and poor patient prognosis. However, p53 mutations alone are not the only responsible for tumor progression: several upstream and downstream molecules of the p53 pathway (MDM2, p19ARF and Bax) are usually altered in human tumors [44]. Nevertheless, studies have failed to correlate p53 mutations with reduced toxicity to anti-cancer agents in some tumors like melanoma [45].

Mutations can vary according to the tumor tissue, and different drugs must be tested to attack different types of cancers. 5-fluorouracil (5-FU), for example, is the most common antimetabolite used for the treatment of colorectal cancer. Researchers have demonstrated that 5-FU exerts its cytotoxicity through induction of apoptosis, but the drug is not completely effective because of an inducible chemoresistance mechanism. 5-FU induced the activation of NF-κB in two colon cancer cell lines [21] and four of five thymidylate synthase inhibitor-resistant colon cancer cell lines were found to overexpress NF-κB [20].

NF-κB is a family of ubiquitous transcription factors that includes p50/p105, p52/p100, RelA (p65), c-Rel, and RelB [46]. It is known that numerous proteins, including C-myc, Cyclin D1, Bcl-2, COX-2, Bcl-xL and Survivin, are all regulated by NF-κB at the transcriptional level and linked to chemoresistance. [11,19,47-50] showed that transfection with adenovirus IκBα super-repressor strongly inhibited constitutive activation of NF-κB and significantly enhanced 5-FU and 5-FU/Folinic acid-mediated growth inhibition.

Many other studies have demonstrated a correlation between NF-κB and chemoresistance in different cancer cells. Chemoresistance in pancreatic cancer, for example, has been associated with activation of NF-κB, and its inactivation leads to cell sensitization to conventional therapeutics [51-53]. Gemcitabine remains as the best treatment available for advanced pancreatic cancer, but this drug alone activates NF-κB, decreasing the apoptosis rate *in vitro*[54,55].

Wang *et al.*[55] showed that escin, a natural mixture of triterpene saponins, increased apoptosis rate in BxPC-3 and PANC-1 cells by downregulating NF-κB, and consequently C-myc, Cyclin D1, Bcl-2, COX-2, Bcl-xL and Survivin. They also showed that the combination of gemcitabine and escin was more effective. Xiao & Wei [56] and Harikumar *et al.*[57] have already reported that escin could reduce the activity of NF-κB. Other works demonstrated that escin exhibited antitumor effects in various cancer cells [58-61] and enhanced the effects of paclitaxel and doxorubicin in human hepatocellular carcinoma cells.

A long-term treatment of cancer cells with a chemotherapeutic drug usually results in cells resistant to the treatment. Barr *et al.*[62] established cell lines resistant to cisplatin with increasing doses of the drug and demonstrated that these cells have increased NF-κB expression and stem cell-like signature. Treatment of resistant cells in murine models with genistein, an Akt/NF-κB inhibitor, sensitizes cells to cisplatin-induced cell death [63].

**Figure 1:**
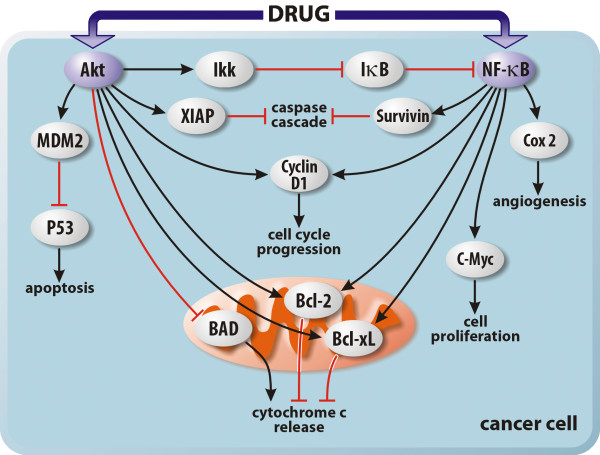
**The role of Akt and NF-κB signaling in drug resistance mechanism of tumor cells.** Several drugs induce the activation of Akt and NF-κB signaling pathways. Akt activation inhibits the P53 and BAD pathways leading to cell survival. Akt also stimulates Ikk, which inhibits IκB and triggers NF-κB signaling pathway. NF-κB activates C-Myc and COX 2 that are mainly involved in cell proliferation and angiogenesis, respectively. Both Akt and NF-κB activate the antiapoptotic proteins Bcl-2 and Bcl-xL, inhibiting the Cytochrome-c release from mitochondria, and Cyclin D1, which contributes to cell cycle progression. Akt and NF-κB also inhibit the caspase cascade by XIAP and Survivin activation, respectively.

Evidences suggest that not only NF-κB, but also PI3K/Akt signaling pathway, is associated with chemoresistance development in cancers (Figure 1). PI3K-Akt pathway is a known regulator of cell survival that controls pro-survival and antiapoptotic proteins such as Cyclin D1, Bcl-2, Bcl-xL and XIAP [64-67].

Many natural products that activate different stages of the cell death cascade are synergistic in combination with effective chemotherapeutic agents. For example, curcumin, the yellow pigment in Indian saffron, potentiates the antitumor activity of various chemotherapeutic agents, including paclitaxel, gemcitabine and cisplatin, in a wide range of cancer cells by suppressing the expression of important antiapoptotic proteins [68-70].

Natural products have been studied in different tumor models due to their effective potential against cancer cells and relatively low cytotoxicity in normal cells [71-73]. Actually, natural products could protect normal cells against pathological changes caused by drugs like doxorubicin [11].

Yang *et al.*[74] found that tectorigenin, a type of O-methylated isoflavone, does not induce potent cell death alone, but significantly sensitizes human ovarian cancer cells to paclitaxel-induced cytotoxicity by inactivating the Akt/IKK/IκB/NF-κB signaling pathway. Flavonoids like tangeretin and genistein showed important chemosensitization of drug-resistant ovarian cancer cells to different agents like cisplatin and taxane drugs, as well as gemcitabine and topotecan. These natural compounds increased cell death by downregulating the PI3K/Akt pathway [75].

PI3K/Akt is another important signaling pathway involved in acquired chemoresistance of many cancers. Akt is also known as protein kinase B (PKB). All of its isoforms (Akt1, Akt2 and Akt3) are phosphorylated (activated) by a phosphatidylinositol 3-kinase (PI3-K) in response to growth factors to promote cell survival [76]. Increased activation of different isoforms of Akt has been associated with different cancers [77-79].

A number of works demonstrated that the Akt pathway is directly related to resistance of cancers against different drugs like sorafenib, trastuzumab and erlotinib [80-82]. The epigenetic control of Akt and NF-κB is important for the establishment of drug resistance. Lin *et al.*[83] found that RUNX3 suppresses Akt1 transcription by directly binding to the Akt1 promoter. Zheng *et al.*[84] showed that methylation of RUNX3 induces activation of the Akt signaling pathway. This mechanism of control would be responsible for inducing docetaxel chemoresistance in human lung carcinoma and the treatment of docetaxel-resistant lung cancer cells with a specific DNA methyl-transferase inhibitor decreased the cell viability.

Different compounds have been used to target the Akt pathway. Liu *et al.*[85] induced apoptosis in gefitinib-resistant lung cancer cells by using benzyl isothiocyanate, which suppressed the activity of Akt/MAPK pathways. Gao *et al.*[86] found that apigenin, a natural flavonoid, could be an adjuvant sensitizer in doxorubicin-resistant hepatocellular carcinoma, once this natural compound inhibited the PI3K/Akt/Nrf2 pathway in the resistant cells.

Anoikis is a type of programmed cell death that is induced by disruption of cell-matrix interactions in epithelial cells, named so by Frisch and Francis [87], although there are reports of the anchorage-dependent cell growth and viability dating back to the 1960’s [88]. This process is an important step for maintaining the balance between cell proliferation and cell death in healthy tissues [89]. Basically, extracellular matrix (ECM) signaling and interactions with epithelial cells determine their correct location, preventing detached cells to colonize different tissues than their own. However, an important aspect of chemoresistance in cancer cells (especially in carcinomas) is resistance to anoikis, which can confer these cells the ability to detach from their original tissue and not only survive but also migrate to secondary sites and invade other tissues, i.e. metastasize [90].

Anoikis activation is associated with the inactivation of pro-survival genes after cytoskeleton rearrangement [91]. In cancer, pro-survival pathways, such as Akt and ERK pathways, are activated to suppress anoikis signaling. Researchers have implicated Akt pathway activation in anoikis suppression during resistance to chemotherapeutic agents as mitoxantrone, cisplatin and 5-FU, but no decreased sensitivity to paclitaxel [92]. In other study, overexpression of CEACAM6 (Carcinoembryonic Antigen-related Cell Adhesion Molecule 6) was associated with chemoresistance to gemcitabine in pancreatic adenocarcinoma [93]. However, Diaz-Monteiro and McIntyre [94] have found that anoikis resistance is not directly related to chemotherapy resistance in osteosarcoma, probably involving distinct activation steps.

Recently, works on miRNAs approached different aspects of anoikis resistance and chemoresistance, i.e., reducing mobility of anoikis-resistant cells as well as increasing their sensitivity to paclitaxel in endometrial and ovarian cancer cells [95]. Even though studies have managed to place intermediate proteins in signaling pathways leading to anoikis resistance, there are many different pathways that culminate in the evasion of anoikis. Hence, much is yet to be discovered to elucidate this process in cancer progression.

### Heat shock proteins in tumor resistance

Other sensitizers that have been studied in potential combinational therapies are the heat shock proteins (HSPs). HSPs are chaperones with cytoprotective role into the cells responsible by proper folding of proteins. HSPs are classified according to their molecular weights in Hsp100, Hsp90, Hsp70, Hsp60, Hsp40 and small HSPs [96]. Among these proteins, Hsp90, 70, 40 and 27 have received special attention in studies that aim to inhibit tumor growth and progression. Hsp70 and Hsp90 are proteins directly involved in refolding proteins; Hsp40 transfers the unfolded protein to Hsp70 by complexing with HIP (Hsp70 interacting protein) and stimulates the ATPase activity of Hsp70; Hsp27 prevents aggregation of unfolded proteins into the cytoplasm [97,98].

Jeong *et al.*[99] demonstrated the association between Hsp90 inhibition and decrease in proliferation of a non-small cell lung cancer (NSCLC) cell line resistant to gefitinib. Other study using NSCLC cells showed that treatment with Hsp90 inhibitor ganetespib induced loss of EML4-ALK gene rearrangement found in this type of tumor and depletion of multiple oncogenic proteins [100]. Hsp90 inhibitor CH5164840 showed antitumor activity on NSCLC cell lines and enhanced the efficacy of erlotinib. The combination of these compounds suppressed ERK signaling in a cell line resistant to erlotinib [101]. Hsp90 inhibition lead to apoptosis induction by mitochondrial pathway in melanoma, cervix, colon, liver and lung cancer cells and induced apoptosis in cells overexpressing Bcl-2 [102].

Hsp70 is currently upregulated in several cancer types and can be induced by drugs that trigger the heatshock pathway signaling. Hsp70 protects normal and tumor cells from death by binding to Bax and Apaf-1 after a stress stimulus [103,104]. A barrier to the complete successful of Hsp90 inhibition in treatment of cancers is that its inhibition increases the Hsp70 expression [105]. Some authors showed that inhibition of Hsp70 alone is few less or ineffective to cause cell death in tumors, nevertheless it could enhance the antitumor effects of other drugs a great coadjuvant in the treatment of cancers [106-108]. The Hsp40 group has a role as co-chaperone for Hsp70 and indirect regulator of Hsp90 and it contains the greatest number of members. In fact, the diversity of structures and functions of the group makes targeting Hsp40 very challenging (for review, see Sterrenberg *et al.*[109]).

Different works showed the relationship between Hsp27 and direct activation of Akt, increasing the cell survival signaling pathway by regulating negatively pro-apoptotic proteins in different models [110,111]. Kim *et al*. [112] demonstrated that inactivation of ERK/p90RSK/HSP27 cascade in SK-OV-3 cells by melatonin enhances cisplatin-induced apoptosis. Hsp27 inhibition by quercetin also reduced the viability of A549 cells when used in combination with cisplatin or gemcitabine when compared to these drugs alone showing the role of Hsp27 in chemoresistance [113]. Other studies showed the antitumor effects of Hsp27 inhibition in combination with other drugs (for review, see McConnell & McAlpine [114].

### Monolayer x 3D cell culture

Cell culture in a monolayer system, also known as two-dimensional culture (2D), does not maintain the same features found *in vivo*. The development of other culture systems are growing up to achieve one that better mimics the *in vivo* cellular features, very important to improve studies about cancer disease, for example, in the evaluation of drug effects in cancer cells [115]. The three-dimensional culture (3D) is a type of culture that increases cell interactions with other cells and with the ECM, which is closer to *in vivo* conditions [116,117].

The increased cell-cell or cell-matrix interactions observed in 3D culture can: a) augment cell differentiation [118-120]; b) change cell signaling in response to ECM compounds [121]; c) modify the gene expression pattern [122,123]; and d) alter the expression of proteins linked to cell adhesion to matrix (integrins) and cell-cell adhesion (cadherins) [124]. The expression of integrin and E-cadherin distribution in spheroids were similar to *in vivo* results [125,126].

There are several models of cell culture in a 3D environment, such as: multicellular spheroids [127,128], microcarrier beads, synthetic (synthetic gels) or natural materials (matrigel, a gel with ECM obtained from mouse sarcoma cells in culture, and type I collagen) that provide cell growth in a three-dimensional system and organotypic explant culture [121].

The 3D culture may be a good model for both basic and applied research. Cancer cells culture in a 3D system is very interesting to study cancer disease, for example, evaluating the effects of drugs in these cells. Cells maintained in a 3D environment are organized in multiple layers that confer a biological barrier to drug diffusion, like small avascular tumor aggregates observed *in vivo*[121]. Fourré *et al.*[129] cultivated fibrosarcoma cells HT-1080 in a 3D culture type with type I collagen and showed that doxorubicin cell penetration took about 1 more hour compared to cells grown in a 2D system. Other works showed similar results with the same drug: Yip & Cho [130] found that cells cultured in the presence of collagen hydrogel had higher cell viability and Millerot-Serrurot *et al.*[131] observed that ECM protected cancer cells from anti-migratory effect of doxorubicin. However, in these cases, the decreased drug penetration was due to mechanical resistance and not cell chemoresistance [132].

Some cell types become more resistant to cell death via apoptosis while they are in contact with other cells or with the ECM, as it occurs in 3D cell cultures. For example, cell adhesion mediated by integrins leads to increased expression of integrin receptors and fibronectin, such as VLA-4, which seems to be related to apoptosis resistance [133,134]. Also, in some 3D models in which cells became polarized after the contact with an ECM similar to the basement membrane, the expression of beta4 integrin was associated with resistance to apoptosis [135]. This type of resistance, also known as CAM-DR (cell adhesion mediated drug resistance), is seen today as a target to anti-cancer therapies [136,137].

Studies have shown that cells are more resistant to drugs when grown in 3D cultures than when they are in monolayer. Longati and co-workers [138] tested the resistance of pancreatic ductal adenocarcinoma cell line (PDAC) in 2D and 3D cultures. The cells are more resistant to gentamicin, CD5, CB7, CB13, Act16412 and GANT61 when they are in 3D cultures. Human ovary cancer cells (SKOV3 cells), when cultured in 3D to mimic ascites, form cell aggregates resistant to paclitaxel [139,140]. This resistance could be due to high expression levels of KLK4 (high tumor kallikrein-related peptidase 4) [140]. Similarly, stem cells isolated from SKVO3 cultures, when cultivated in a 3D environment with basement membrane extract scaffold, are resistant to docetaxol, cisplatin, carboplatin and 5FU. In cells grown in this model of 3D culture, the expression of ABCB1 and ABCG2 are increased and could be related to resistance to the drugs tested [141]. Lung cancer cells also become resistant to bortezomib when in 3D cultures [142], as well as MCF-7 cells which become resistant to 5-FU in specific stages of spheroid formation [143].

Some genes related to drug resistance in 2D cultures are over expressed in 3D cultures and could be involved with drug resistance in these models. Among these genes, we can mention BCL-2 family members, ABCG2 and ABCB1, CP78 and KLK4. Other interesting hypothesis for drug resistance in 3D cultures is related to the increased cell adhesion and matrix elements synthesis in these models, making it difficult for drugs to penetrate in the spheroids and reach all the cells [138]. However, some studies show that certain drugs such as doxorubicin can penetrate in spheroids and be incorporated in cells nuclei within these large structures. In this case the drug retention does not depend on MDR1 bombs, but the resistance seems to be related to the expression of Bcl-2 family members [31].

Fourré and co-workers [129] also show in collagen rich models that doxorubicin and anthracyclins take more time to be detected in cell nuclei and that in these cases it takes longer treatments to reach similar cellular responses to those seen in 2D cultures.

Nirmalanandhan and co-workers [144] tested 10 different drugs in lung cancer (A549) and bronchioalveolar cancer (H358) cells cultured in 2D and 3D models with type I collagen. When tested in H358 cells, 8 of the 10 drugs needed different concentrations to reach the same effects in 2D and 3D. In A549 cells 7 of the 10 drugs showed similar effects but in different concentration. The results depend on the cell line and on the drug class, and show that more studies should be done to determine if the mechanisms of chemoresistance in 3D models share the same features of the mechanisms observed in 2D cultures. Moreover, it is important to evaluate if the cell responses to drugs in 3D are more similar to what happens *in vivo* than those observed in 2D cultures, making it a new way to test drugs and to evaluate chemoresistance. A summary of studies with drug resistance in 2D and 3D cell cultures is presented in Table 1.

**Table 1:** Effects of some drugs and cancer cell mechanisms of drug resistance in monolayer and in three-dimensional cell cultures

**Drug**	**Cell type**	**Effects in 2D and 3D cultures**	**Processes related to chemoresistance**	**Reference**
Doxorubicin/Paclitaxel/Tamoxifen/	MCF-7 (breast carcinoma)	Antiproliferative effect increased in 2D; cells more resistant to drugs in 3D.	Increased ECM production in 3D models, difficulting drug diffusion.	Horning *et al.*[143]
AG1478	SW-480, HT-29, DLD-1, LOVO, CACO-2. COLO-205, COLO-206F (Colorectal Cancer Cell Lines)	Cell viability decreased in a dose-dependent way in 2D and 3D, but more clearly in 2D.	Cells in 3D showed decreased expression of EGFR. ECM signaling can be involved too.	Luca *et al.*[98]
5FU/ Docetaxel/ Cisplatin/Carboplatin	SKOV-3 cell line (epithelial ovarian cancer). Stem cells were selected from these cultures.	Antiproliferative effect increased in 2D. Cells in 3D models more resistant to drugs.	Decreased apoptotic induction in 3D. Increased expression of ABCG2 e ABCB1 in 3D.	Chen *et al.*[116]
Paclitaxel	SKOV-3 (epithelial ovarian cancer)	Increased expression of KLK4 favors multicellular aggregates formation and these are more resistant to drug.	Increased expression of KLK4.	Dong *et al.*[115]
Doxorubicin/ Cisplatin	SW1353, CH2879, JJ012, OUMS27 (chondrosarcoma cell lines)	In 3D models doxorubicin can reach cell’s nuclei but cells are more resistant to the drug.	Drug penetration is not dependent on MDR activity. Bcl-2 members are important to resistance.	Van Oosterwijk *et al.*[29]
Taxol/Cisplatin	HEY, A2780, SKOV3, OVAC429 (human ovarian cancer)	Cells cultured in 3D are more resistant to taxol treatment.	Cells from some cell lines did not arrested in G2-M after taxol treatment.	Frankel *et al.*[144]
Doxorubicin	HepG2 (human hepatocellular liver carcinoma) and 3T3-J2 (fibroblasts murine stromal cells)	Increased cell viability in 3D. 3D heterospheroids are more resistant than 2D and homospheroid models.	Stromal fibroblasts and collagen hydrogel culture system provides more resistance.	Yip & Cho [105]
Doxorubicin	HT1080 (human fibrosarcoma)	In 2D doxorubicin decreased cell migration, and in 3D the drug did not affeted cell migration.	EMC proteins in a 3D configuration are able to protect cancer cells from the antimigratory effect of doxorrubicin. Environment-mediated drug resistance.	Millerot-Serrurot [106]
Docetaxel/Cisplatin/5-FU/Gemcitabine/ Camptothecin	H460, A549, H1650 (lung cancer)	All drugs showed increased IC-50 in 3D.	Caspase-3 is decreased in 3D. Drugs could not penetrated into cells in 3D and apoptosis was decreased.	Godugu *et al.*[107]

The table shows authors that compared effects in 2D and 3D cell cultures in the same article.

### Cancer stem cells

The concept of cancer stem cell (CSC) was stated based on the organization of multicellular organisms presenting somatic stem cell populations that give rise to committed progenitors which are able to differentiate into mature cells. Normal cellular hierarchy comprises stem cells that progressively generate more restricted progenitor cells, yielding all the mature cell types that constitute a particular tissue. Cancer would simulate organ development, exhibiting a similar hierarchy with different cell populations, including the CSCs, associated to high drug resistance.

In the strict sense, CSCs and tumor initiating cells (TICs), i.e., cells that acquired the tumor promoting mutations are conceptually different. CSCs (and not other tumor cells) would be the only cells capable of sustaining tumorigenesis due to their self-renewal and asymmetric division abilities. TICs are defined as cells capable of initiating a tumor in immunocompromised mice [145]. However, the terms CSCs and TICs have being indistinctly used to refer to the small cellular subpopulation (0.01-1% of total tumor cells) first described in leukemia and then in breast cancer and others solid tumors [16,17,146,147]. These cells are able to induce cancer when transplanted to immunodeficient mice, have drug resistance and self-renewal ability. It is believed that the resistance of CSCs to currently used chemotherapeutics is a major contributing factor in cancer recurrence and later metastasis development.

According to their phenotypes, CSCs can be identified and isolated by means of 4 main methodologies: a) cell sorting by flow cytometry using specific cell surface markers [148,149]; b) assessment of aldehyde dehydrogenase (ALDH) activity [150]; c) cell sorting of side-population (SP) phenotype by Hoechst 33342 exclusion [151]; d) spheres isolation, since CSCs are able to form floating colonies from a single cell more efficiently than their progeny [152] and to grow as spheres in non-adherent culture conditions [153].

The most commonly used surface markers are CD44+ and CD133+ [17,146]. CSC phenotype in leukemia was associated with CD44+/CD38- cells [16]. CD44 is a transmembrane glycoprotein believed to be activated in a wide range of tumors in which it plays a critical role in cancer cell adhesion, migration, invasion and survival [154]. It is a multifunctional cell surface adhesion molecule associated with cell-cell and cell-matrix interaction. CD44+ has identified cells with the ability of give rise to new tumors *in vivo*, in different types of cancer. Patient samples of head and neck squamous cell carcinoma (HNSCC), for example, contain a heterogeneous population of cancer cells and the small subpopulation CD44+ contained most of the CSCs, evidenced by its tumorigenic potential in immunodeficient mice [155].

CD133 (prominin-1 or AC133) was originally described in human hematopoietic stem cells and has subsequently been used as a marker to isolate CSCs from many tumor types. It is a member of the pentaspan transmembrane glycoprotein family involved in a variety of cellular activities. CD133 is found to be selectively localized in microvilli and other plasma membrane protrusions irrespectively of cell type and interact with membrane cholesterol. Wnt, Notch, TGFβ1, Line-1 and methylation regulate its expression. CD133 is involved in energy metabolism and in autophagy, which are beneficial for the survival of cancer stem cells.

ALDH activity is an important functional marker of normal and malignant stem/progenitor cells. ALDHs contribute to drug resistance through detoxification of many cytotoxic agents provided that aldehydes are generated by several metabolic processes (reviewed by Marchitti *et al.*[156]). Increased ALDH activity in hematopoietic stem cells, for example, contributes to metabolize and detoxify cyclophosphamide [157]. The ALDH family of enzymes comprises 19 isoforms that can be found in different cell compartments: nucleus, cytoplasm or mitochondria. In a retrospective analysis of breast cancer patient samples, ALDH1A1, but not ALDH3A1, expression was found to be predictive of tumor responsiveness to cyclophosphamide and other oxazaphosphorines treatment [158]. In support of this potential role for ALDH in CSC resistance to chemotherapeutics, CSC enrichment was observed in colorectal cancer xenograft tumors after cyclophosphamide treatment, and this was correlated with enhanced ALDH1A1 expression and enzymatic activity.

Antibodies against the ALDH enzyme family are available, but the vast majority of studies have used cell-sorting techniques to enrich for cells expressing these enzymes. Live cells expressing high ALDH activity are usually identified by the Aldefluor assay and sorted by fluorescence-activated cell sorting. This approach was used by Cheung *et al.*[159] in one of the first studies isolating ALDH+ cells from acute myeloid leukemia. ADLH+ enriched cell population was similarly isolated from breast cancer [160]. In both studies, the isolated cells presented self-renewal ability and high tumorigenic potential. ADLH+ cells with CSC phenotype were isolated from several hematopoietic and solid tumors including lung, liver, bone, colon, pancreatic, ovarian, head and neck, and prostate cancers.

The aldefluor activity specific for the CSCs of these cancers has been attributed to ALDH1A1 and so prognostic studies have been targeted to this isoform. However, Marcato *et al.*[161] claim that ALDH1A3 and other ALDH isoforms activities contribute to aldefluor positivity.

Additionally, ALDHs participate in ester hydrolysis and act as antioxidant. Enzymatic aldehyde dehydrogenase activity of some specific isoforms is important for the preservation of undifferentiated stem cells, by interfering with the biosynthesis of endogenous retinoic acid (RA) through the oxidation of all-trans-retinal and 9-cis-retinal. The cytosolic isoform ALDH1A1, associated with metabolism and detoxification of cyclophosphamide, plays a role in the differentiation of several cell types through the oxidation of retinal to RA [156].

RA modulates biological processes like cell proliferation, differentiation, cell cycle arrest and apoptosis. All-trans-RA is used to treat acute promyelocytic leukemia, since it induces differentiation of immature leukemia blasts into terminally differentiated granulocytes, leading to a clinical remission in approximately 90% of patients. Based on these results, retinoic acid effects are being studied in other cancers and cancer cell lines. The combined use of RA (0.1 μM) and cAMP (1 mM), an important second messenger, improves the responsiveness of hepatocarcinoma cell line (HTC) to RA treatment. RA and cAMP were effective in inhibiting the proliferation of HTC cells independently of combined use. However, treatment with RA and cAMP increased E-cadherin, Cx26, Cx32 and Ser9-GSK-3β (inactive form) expression while the expression of Cx43, Tyr216-GSK-3β (active form) and phosphorylated ERK decreased, showing that the combined use of RA and cAMP is more effective in inducing differentiation [162].

The use of the vital dye Hoechst 33342 exclusion as a method to isolate normal hematopoietic stem cells was proposed by Goodell *et al.*[163]. The method defines an easily identifiable and highly reproducible small cell population (0.1% of bone marrow cells), presenting stem cell phenotype. The Hoechst-exclusion SP assay has the advantage of measuring a functional parameter of the cells. Widely used in hematological malignancies, the methodological approach requires additional steps such as enzymatic cell disaggregation for solid tumor samples analyses [164]. Both normal and cancer stem cells express the ABC transporters. The ABC domain of these transmembrane proteins allows ATP binding and hydrolysis, and the ABC protein can function as receptor, channel and multidrug transporter, participating in the efflux of small molecules. These pumps detoxify cells through the efflux of cytotoxic agents, being responsible for the exclusion of the dye Hoechst 33342.

SP cells were isolated and characterized in most human cancers including HNSCC, bladder, ovarian, pancreas, lung, hepatocellular carcinomas, osteosarcoma and Ewing’s (for review, see Tirino *et al.*[164]). SP cells were consistently shown to represent a stem cell-enriched population. Compared to non-SP cells, a smaller number of SP cells are able to grow as tumors when injected in immunodeficient (NOD/SCID) mice.

Breast cancer cell lines, like MDA-MB-231 and MCF-7, show anoikis-resistance in drug treatments with doxorubicin. The SP cells fraction in the anoikis-resistant cancer cells seems to be higher than the parental cells [165]. There are reported mechanisms that contribute to SP chemoresistance including relative quiescence, expression of ABC transporters and/or MDR1, a more efficient DNA-repair capability, and the elevated expression of anti-apoptotic proteins.

The high tumorigenic efficiency of SP cells is associated with drug resistance and with the presence of other CSC markers, such as ALDH+, CD133+ or CD44+. He et al. (2013) proposed the phenotypical modulation of CSCs, which involves the conversion of SP to non-SP cells (and vice versa), to be under PI3k/AKT and β-catenin /CBP signaling pathway. Beta-catenin accumulation enhanced the transition from non-SP to SP phenotype, and siRNA against any of the downstream signals abrogated the conversion of non-SP to SP cells in breast and bladder cancer cell lines.

Other method for CSC isolation was based on the observations of Reynolds *et al.*[166] that some cells of the central nervous system were able to grow in suspension when plated on non-adherent surface, forming structures named spheres or neurospheres. These floating colonies were able to self-renewal, once when enzymatically dissociated, they originated several new spheres. Their stem cell phenotype was confirmed by the ability to originate different cell types under adequate stimulation (astrocytes, neuron or oligodendrocytes). The floating sphere formation is consequence of the ability to grow independently of surface anchorage and resistance to anoikis associated with high clonogenicity, features shared by both normal and cancer stem cells.

Spheres were grown from different human cancer samples and cancer cell cultures and they consisted mainly of CSCs (review in Alamgeer*et al.*[167]). SCLC and NSCLCCD133+ cells, when submitted to long-term culture as spheres, could modify their phenotype to CD133- cells [153]. The phenotype modulation of CSCs is important to define more efficient therapies. MCF-7 cell line long-term spheroids showed high degree of cell differentiation, organizing duct-like structures [119].

The CSC model represents a very important tool in cancer biology, especially in relation to the problem of drug resistance. CSC/TIC cells may exist independently of the described markers and the cellular plasticity may be much more relevant. Nevertheless, the current identification of markers and pathways is already underpinning some novel developments in therapeutic strategies for patients with cancer.

## Conclusions

The resistance to chemotherapy was described in cancer longtime ago, being responsible for most of treatment failures. Remarkable progress has been achieved in understanding the tumorigenesis and cancer progression molecular mechanisms, contributing to the elucidation of some aspects associated with lack of response to treatment. Traditionally, it has been proposed that genetic instability would be responsible for generation of drug resistant tumor cells, according to the clonal theory of cancer development. Alternatively, cancer cells present different mechanisms of drug resistance including innate mechanisms that operate at stem cells and functional responses that result in modulation of intracellular signaling pathways. The major contribution of the study of the drug resistance mechanisms is the definition and implementation of more effective, and perhaps personalized, treatment protocols. Multiple sensitization using natural products and combined protocols are currently in use to reduce or deplete the resistance; however the establishment of 3D cell cultures, a system closer to the *in vivo* tumor, would represent a valuable tool to cancer treatment.

## Abbreviations

2D: Two-dimensional; 3D: Three-dimensional; 5-FU: 5-fluorouracil; ABC: ATP-binding cassette; Akt: Protein kinase B; ALDH: Aldehyde dehydrogenase; CSC: Cancer stem cell; CSCs: Cancer stem cells; ECM: Extracellular matrix; HNSCC: Head and neck squamous cell carcinoma; IκB: Inhibitor of nuclear factor κB; IKK: IκB kinase; KLK4: High tumor kallikrein-related peptidase 4; MDR: Multidrug resistance; MiRNAs: Micro RNAs; MRP1: Multidrug resistance associated protein 1; NF-κB: Nuclear factor κB; P-gp: P-glycoprotein; PI3K: Phosphatidylinositol 3-kinase; RA: Retinoic acid; SP: Side-population; TICs: Tumor initiating cells.

## Competing interests

The authors declare that they have no competing interests.

## Authors’ contributions

ELON and GMM-S were the main authors of the manuscript; AAM, BAC, BR-S, CL, ELON, JHN, MSU, MMS, PR-T and GMM-S collected and studied the bibliography and drafted the manuscript; ELON and GMM-S revised the manuscript critically for important intellectual content. All authors read and approved the final manuscript.
